# Visual implications of digital device usage in school children: a cross-sectional study

**DOI:** 10.1186/s12886-019-1082-5

**Published:** 2019-03-12

**Authors:** Parul Ichhpujani, Rohan Bir Singh, William Foulsham, Sahil Thakur, Amtoj Singh Lamba

**Affiliations:** 1 Department of Ophthalmology, Government Medical College and Hospital, Sector 32, Chandigarh, India; 2 Massachusetts Eye and Ear Infirmary, Department of Ophthalmology, Harvard Medical School, Boston, USA; 3 Institute of Ophthalmology, University College London, London, UK

**Keywords:** Eyestrain, Digital devices

## Abstract

**Purpose:**

To evaluate the use of digital devices, reading habits and the prevalence of eyestrain among urban Indian school children, aged 11–17 years.

**Methods:**

The study included 576 adolescents attending urban schools who were surveyed regarding their electronic device usage. Additional information on the factors that may have an effect on ocular symptoms was collected.

**Results:**

Twenty percent of students aged 11 in the study population use digital devices on a daily basis, in comparison with 50% of students aged 17. In addition to using these devices as homework aids, one third of study participants reported using digital devices for reading instead of conventional textbooks. The majority of students preferred sitting on a chair while reading (77%; 445 students), with only 21% (123 students) preferring to lie on the bed and 8 students alternating between chair and bed. There was a significant association between the students who preferred to lie down and those who experienced eyestrain, as reported by a little over one fourth of the student population (27%). Out of 576 students, 18% (103) experienced eyestrain at the end of the day after working on digital devices.

**Conclusions:**

The increased use of digital devices by adolescents brings a new challenge of digital eyestrain at an early age. Our study reports the patterns of electronic device usage by school children, evaluates factors associated with eyestrain and highlights the need for further investigation of these issues.

## Introduction

Asthenopia is clinically defined as a subjective sensation of visual fatigue, eye weakness or eyestrain. It results from imbalance of extraocular muscles, uncorrected refractive errors, accommodative impairment and improper lighting [1]. Patients suffering from asthenopia present with excessively watery eyes, double vision, blurred vision, itching, sore eyes, headache, dry eye sensation and redness [2]. A recent meta-analysis pooled the prevalence of asthenopia in children at 19.7% [3]. Individuals who spend long periods looking at computer displays have intense accommodation and extraocular muscle strains, and often exhibit asthenopia [4]. In the current era, children (even toddlers) are growing up with touchscreen technology at their fingertips. It is reasonable to speculate that the increased use of mobile phones, tablets etc., may contribute toward the rising prevalence of asthenopia in the young. However, our knowledge of eyestrain in the young is currently limited by a scarcity of data investigating the association of asthenopia with behavioral risk factors. In view of the sparse literature on the subject of digital eyestrain in school children, the present study was conducted to assess the prevalence of eyestrain and its relation to digital device use in Indian school children aged 11–17 years.

## Materials and methods

**Fig. 1:**
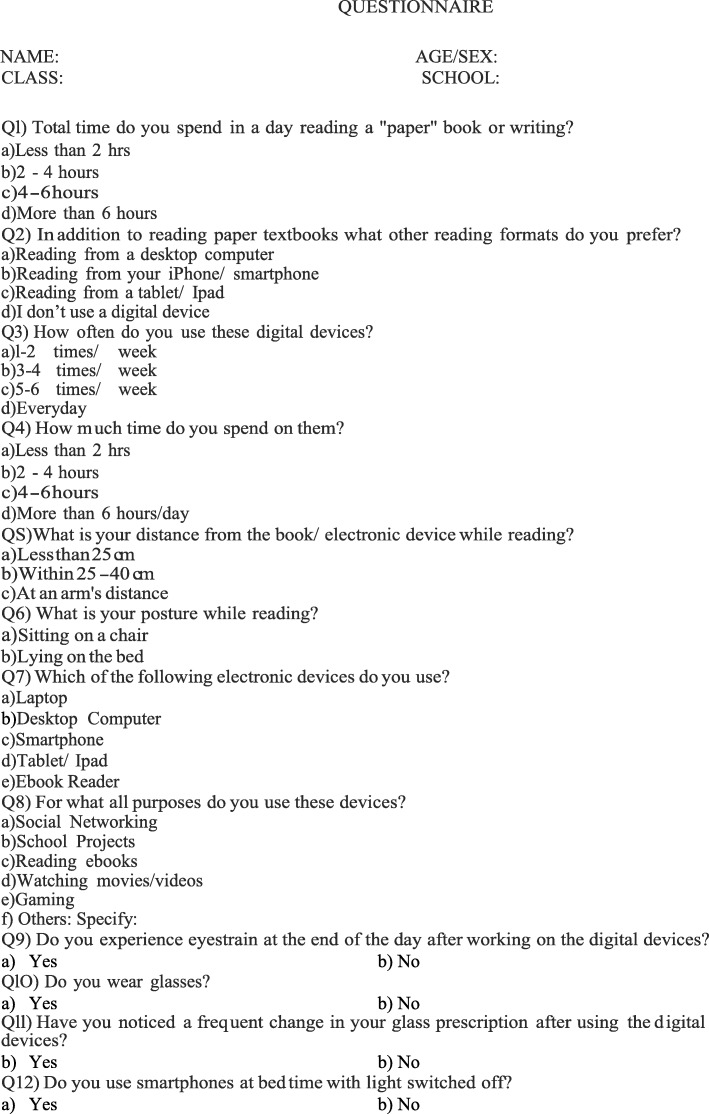
Questionnaire used in the study

A pilot, cross-sectional study was carried out with students attending sixth to twelfth grades at Chandigarh private schools between April and May 2016. The study population was selected from the lists of students provided by the schools. A basic ocular examination using torchlight was performed for each participant. The cover test was performed to exclude patients with squint and a small fixation target was used to assess convergence insufficiency. The students with ocular diseases like corneal scars, cataract, ptosis, manifest squint or treatment patches for amblyopia were excluded from the study. A questionnaire designed by the investigators was administered to the students with the assistance of the on-site research team. As eyestrain or asthenopia is a symptom complex that can present as eye pain, blurring, itching, watering and headache, the term was explained to the children before questionnaire administration. Children were asked questions about the electronic devices they used, the average number of hours of use in a day as well as the distance and posture while reading. Additional information on factors that may influence ocular symptoms was also collected; such as the use of glasses, frequent changes in glasses prescription and the use of smartphones at bedtime with lights switched off (Fig. 1).

### Statistical analysis

SPSS software (Version 21.0. Armonk, NY: IBM Corp.) was used for statistical analysis. Socio-demographic variables like age and gender, class in which they are studying etc. were recorded as explanatory parameters. The types of electronic devices used, time spent on them and smartphone use at bedtime with lights switched off were taken as exposure variables, eyestrain and frequent change in glass prescription were recorded as outcome variables. A descriptive analysis of all the explanatory, and outcome parameters was done. All the recorded categorical variables were presented in frequencies, and percentages. The Chi square test was used to assess association between explanatory and outcome parameters, and the *P* value was calculated.

### Observations and results

The study included 576 students, 60.6% (349) males; 39.4% (227) females. The mean age of the participants was 13.7 years and the median age was 14 years.

#### Type of digital devices used:

Among different electronic devices, 58.3% (336 students) used a smartphone, 37.3% (215 students) used a tablet/ phablet/ iPad, 35.8% students (206) used a laptop, 23.8% (137) used a desktop computer and 9% (52) used an eBook Reader device for reading.

#### Purpose of the digital device used:

Sixty six percent students (66.7%; 384) used it for school projects, 43.6% (251) used it for gaming purposes, 35.6% students (205) used it for social networking, 30.4% (175) used it for reading eBooks and 29.5%(170) used it for watching movies/videos.

#### Reading habits:

- **Reading habits on a digital device**: Close to one-third of the student population used a

digital device for reading. 38.4% (221) used an iPhone/Smartphone, 36.1% (208) used an iPad/Tablet and 27.8% students (160) used a desktop/laptop as preferred reading device.

b) **Reading distance:** More than 50% of students (322; 55.9%) kept their books/electronic devices at a distance of 25 cms-40 cms, 27.4% (158) kept it at an arm’s length while reading and 16.7% (96) kept their books at a distance of less than 25 cms. Table 1 shows the distribution of reading distance from books by age. There was no statistically signification relationship between the age of the students and distance of the book while reading (χ^2^ = 17.93, *P* = 0.118).
c) **Reading positions:** The majority of students preferred sitting on a chair while reading (77.3%; 445) and only 21.4% (123) lay on the bed while 1.4% (8) either sit or lay on the bed (both) while reading.
d) **Time Spent on reading a book and using a digital device**:
  ***Time spent on book/ paper text reading:*** Out of 576 students, 47.4% (273) spent 2–4 h a day, 34.4% (198) spent less than 2 h a day either reading or writing, 13% (75) spent 4–6 h a day and only 5.2% (30) spent more than 6 h in a day reading a “paper book” or writing in addition to time in school. Table 2 shows the distribution of students and the time spent reading from a paper/writing or a digital device by age. With increase in age there was as statistically significant increase in the time spent by them in reading/writing from a paper book (χ^2^ = 46.95, *P* < 0.001). There was also a difference in time spent (> 6 h) by the students at the age of 15 (1.9%) than those at the age of 16(48.5%).
  ***Time spent on digital device reading:*** However, in terms of time spent on digital devices each day, 38.9% (224) spent less than 2 h a day, 43.6% (251) spent 2–4 h in a day, 14.2%(82) spent 4–6 h and 3.3% (19) spent in excess of 6 h each day using digital devices. With increased age there was a statistically significant increase in the time spent on digital devices (χ^2^ = 41.55, *P* < 0.001). However we have to keep in mind that the population distribution was unequal across the age groups.

**Table 1:** Age wise distribution of reading distance from a book/digital device

		Distance from Book			Total
		Less than 25 cms.	25 cms. - 40 cms.	At an arm’s length	
Age (In years)	11	9 (12.9%)	32 (45.7%)	29 (41.4%)	70 (100%)
	12	23 (16.0%)	80 (55.6%)	41 (28.5%)	144 (100%)
	13	31 (20.5%)	79 (52.3%)	41 (27.2%)	151 (100%)
	14	17 (14.2%)	73 (60.8%)	30 (25.0%)	120 (100%)
	15	12 (23.1%)	29 (55.8%)	11 (21.2%)	52 (100%)
	16	4 (12.1%)	24 (72.7%)	5 (15.2%)	33 (100%)
	17	0 (0.0%)	5 (83.3%)	1 (16.7%)	6 (100%)

**Table 2:** Time spent reading a ‘paper book’ or a digital device (DD)

Age (In years)		Time Spent				Total
		Less than 2 h.	2–4 h.	4–6 h.	More than 6 h.	
11	Book	23 (32.9%)	37 (52.9%)	9 (12.9%)	1 (1.4%)	70 (100%)
	DD	43 (61.4%)	23 (32.9%)	2 (2.9%)	2 (2.9%)	
12	Book	50 (34.7%)	74 (51.4%)	18 (12.5%)	2 (1.4%)	144 (100%)
	DD	69 (47.9%)	61 (42.4%)	13 (9.0%)	1 (0.7%)	
13	Book	54 (35.8%)	77 (51.0%)	16 (10.6%)	4 (2.6%)	151 (100%)
	DD	46 (30.5%)	73 (48.3%)	27 (17.9%)	5 (3.3%)	
14	Book	48 (40.0%)	54 (45.0%)	13 (10.8%)	5 (4.2%)	120 (100%)
	DD	35 (29.2%)	52 (43.3%)	26 (21.7%)	7 (5.8%)	
15	Book	16 (30.8%)	25 (48.1%)	10 (19.2%)	1 (1.9%)	52 (100%)
	DD	15 (28.8%)	26 (50.0%)	8 (15.4%)	3 (5.8%)	
16	Book	4 (12.1%)	5 (15.2%)	8 (24.2%)	16 (48.5%)	33 (100%)
	DD	13 (39.4%)	15 (45.5%)	4 (12.1%)	1 (3.0%)	
17	Book	3 (50%)	1 (16.7%)	1 (16.7%)	1 (16.7%)	6 (100%)
	DD	3 (50.0%)	1 (16.7%)	2 (33.3%)	0	

#### Frequency of digital device usage:

Slightly less than half (278, 48.3%) of students used digital devices every day, 24% (138) used them 3–4 times a week, 15.1% (87) used them 1–2 times a week, and 12.7% (73) used these digital devices 5–6 times a week. With increased age there was a statistically significant association with increased digital device use in a week (χ^2^ = 39.55, *P* < 0.001). As the age increases the daily use of these devices also increases, consistent with only 20% students at the age of 11 using them every day in comparison to 50% students at the age of 17 using them every day. Table 3 shows the distribution of students by age and the frequency of digital device use.

**Table 3:** Frequency of using the digital devices

		Digital Device Usage				Total
		1–2 times a week	3–4 times a week	5–6 times a week	Everyday	
Age (In years)	11	23 (32.9%)	20 (28.6%)	13 (18.6%)	14 (20%)	70 (100%)
	12	30 (20.8%)	46 (31.9%)	15 (10.4%)	53 (36.8%)	144 (100%)
	13	17 (11.3%)	30 (19.9)	18 (11.9%)	86 (57.0)	151 (100%)
	14	10 (8.3%)	26 (21.7%)	11 (9.2%)	73 (60.8%)	120 (100.0%)
	15	3 (5.8%)	10 (19.2%)	11 (21.2%)	28 (53.8%)	52 (100.0%)
	16	3 (9.1%)	5 (15.2%)	4 (12.1%)	21 (63.6%)	33 (100%)
	17	1 (16.7%)	1 (16.7%)	1 (16.7%)	3 (50%)	6 (100%)

#### Eyestrain and smartphone addiction

Out of 576 students, 17.9% (103 students) experienced eyestrain at the end of the day after working on the digital devices (Table 4). Although 36.1% students (208) wore glasses, only 13.9% (80) reported a change in their glass prescription after using these electronic devices. However, there was statistically significant increase in the frequency of patients experiencing eyestrain after reading from a paper book for a prolonged period of time (χ^2^ = 8.28, *P* = 0.040), as close to one fourth of the population (23.3%) suffered from eyestrain after reading for more than 6 h. The question regarding eyestrain with paper books was asked to the students as an extended question to Question No 9. The difference in eyestrain between the students who chose to read on paper compared to those who read on digital devices cannot be clearly delineated by our study, as students who read on paper also use digital devices for playing games, surfing the internet or social media; therefore the influence of digital device use as a potential confounding factors cannot be totally negated.

**Table 4:** Time Spent in reading a paper book versus a digital device and eyestrain

Time Spent (Hours)		Eyestrain		Total
		Yes	No	
< 2	Books	46 (23.2%)	152 (76.8%)	198 (100.0%)
	Digital Devices	40 (17.9%)	184 (82.1%)	224 (100.0%)
2–4	Books	42 (15.4%)	231 (84.6%)	273 (100.0%)
	Digital Devices	45 (17.9%)	206 (82.1%)	251 (100.0%)
4–6	Books	8 (10.7%)	67 (89.3%)	75 (100.0%)
	Digital Devices	14 (17.1%)	68 (82.9%)	82 (100.0%)
> 6	Books	7 (23.3%)	23 (76.7%)	30 (100.0%)
	Digital Devices	4 (21.1%)	15 (78.9%)	19 (100.0%)
Total		103 (17.9%)	473 (82.1%)	576 (100.0%)

**Fig. 2:**
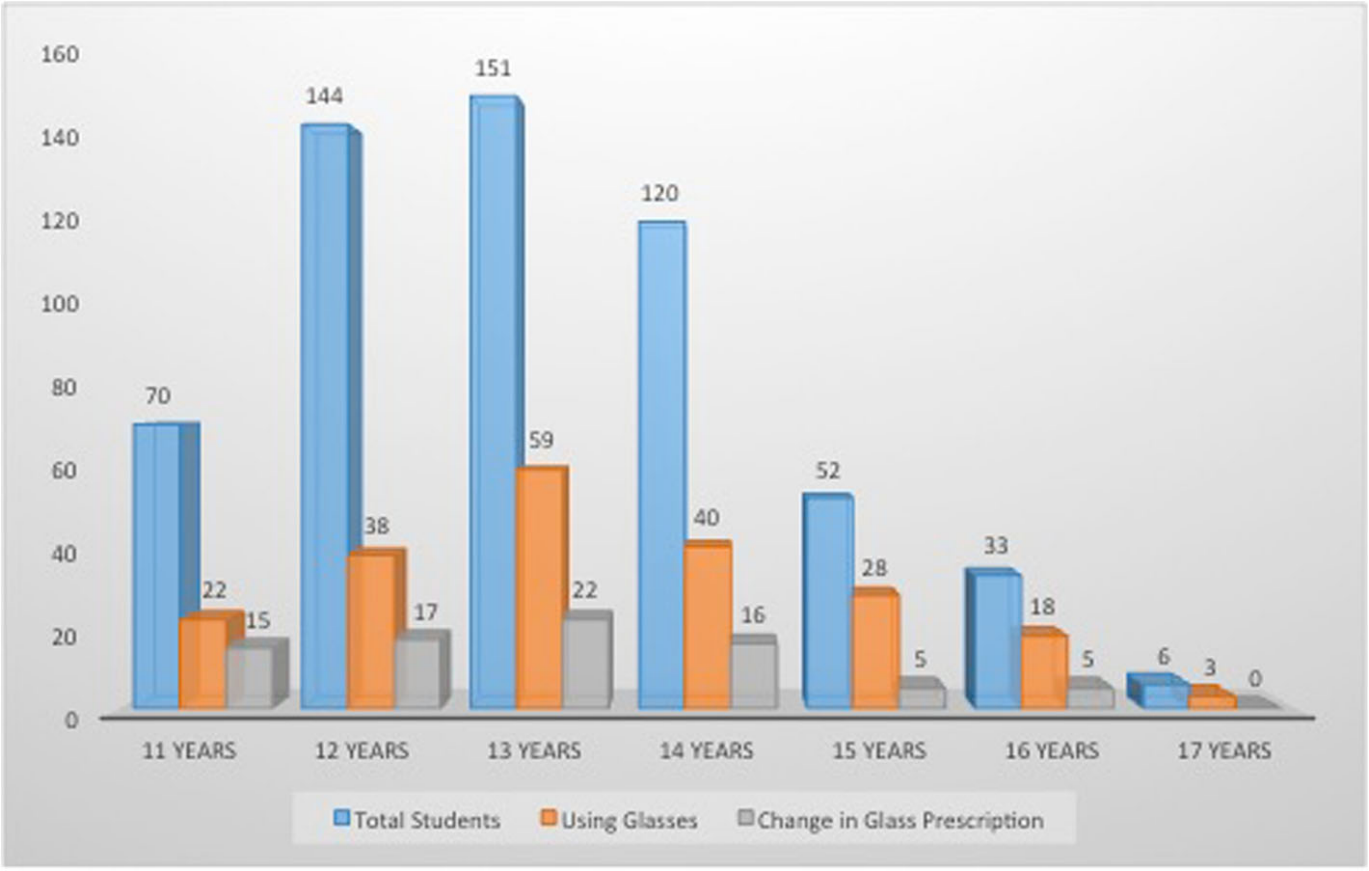
Use of digital devices with associated change in eyeglass prescription

The ratio of those wearing spectacles to those not wearing spectacles in the age group of 11–14 was around 1:3 while the same ratio in the age group of 15–17 was around 1:1. However, there was no statistically significant relation between the age of the students and change in glass prescription (χ^2^ = 5.74, *P* = 0.452). However, the frequency of eyeglass prescription change was higher in those using desktops/laptops. (Fig. 2). Also, there was a statistically significant increase in the use of smartphones with increased student age (χ^2^ = 16.08, *P* < 0.001). In the age group of 11–12 a little less than half the student population (45.3%) used a smartphone while in the age group of 14–16 the smartphone usage significantly increased and close to three-fourths of the student population (72.1%) was using smartphones. Approximately one fifth of students, 19.3% (111), used their smartphones at bedtime with lights switched off. There was also a significant increase in the use of smartphones at bedtime with the lights switched off with increasing student age (χ^2^ = 18.05, *P* < 0.001).

## Discussion

Previous studies have reported the prevalence of eyestrain in children. Ip et al. conducted a comprehensive study evaluating 1448 children, aged 6 years [5]. The investigators estimated 12.6% prevalence of asthenopia in the group. 82% of the children presenting with typical eye fatigue symptoms had normal ocular examination [5]. A study by Abdi evaluated 216 children between the ages of 6 and 16 years, and found 23.1% to be asthenopic [6]. The children had symptoms related to refractive errors, low visual acuity, and accommodative insufficiency [6]. Another study evaluated 72 children, aged 5–9 years, reporting an estimated asthenopia prevalence of 26.4% [7]. Tiwari et al. evaluated children working in the stone polishing and shoe-making industries in India, in order to evaluate the prevalence of asthenopia in minor workers [8, 9]. The control groups used in both studies did not comprise working children, and prevalence of 24.1 and 12.4% were reported respectively [8, 9]. Vilela et al. subsequently reported a 24.7% prevalence of asthenopia in 964 Brazilian school children [10]. The prevalence reported by our study (17.9%) closely matches the pooled prevalence figure of 19.7% determined by a recent meta-analysis of the available studies [3]. However, none of the studies included in the meta-analysis investigated the effect of the use of digital devices by school children, and the possibility that these devices may be contributing towards the ocular symptoms [5–9].

### Reading distance

Reading distance influences the magnitude of symptoms experienced by those using digital devices. The optimum focus distance for reading and writing is 30–40 cm from the eyes. Ideal focus distance is greater for computer viewing, as compared to reading and writing. It is suggested that there is lesser eyestrain when the computer monitor is 50–70 cm away from one’s eyes [11]. Smaller digital devices such as mobile phones are usually held at a distance of 20–30 cm from the eyes, fostering conditions for digital eyestrain. Long et al. recently reported that viewing distances are closer and the resulting eyestrain symptoms are greater after reading for 60 min from a smartphone [12]. In the present study, however, more than half of the students (56%) maintained an ideal reading distance.

### Postural variations and musculoskeletal symptoms

Improper posture leads to excessive straining of eyes and hunching of the back leading to pain in the neck and back muscles. Previous authors have attributed this to incorrect posture and excessive usage of digital devices [13–15]. In our study, we didn’t find any relationship between eyestrain and posture. Of note, most students (77%) preferred sitting on a chair, while the remaining students preferred to lie down while reading or using digital devices.

### Time spent using digital devices

The present study also found that the time spent by students on digital devices each day consistently increases as age increases. It is advised that adolescents should not have screen time for more than two hours a day [15]. This guidance can be challenging for teenagers to follow, particularly since homework frequently requires computer time. Previous studies suggest that the total weekly time spent by adolescents working on computers ranges from 80 to 840 min [15–21]. The present study showed that approximately half of the 13–16 year old students (46.6%) spent 840–1680 min per week using digital devices. This observation is notable, given that previous studies have documented the association of a wide array of health complaints with excessive use of such devices [15, 17, 22]. Accelerated myopia is just one of a plethora of health complaints associated with excessive screen time [23]. Indeed, a recent study showed that children with diagnosed asthma had 1.6 times higher odds of excessively playing computer games as compared to healthy children (95% CI: 1.11–2.30) and children with learning disabilities had 1.7 times higher odds of risky use of the internet (95% CI: 1.19–2.45) [24]. Excessive screen time may be due to numerous factors including the overuse of technology in school as teaching aids, an increasing burden of homework and unrestrained recreational time (surfing the internet, social networking, playing video games and watching movies).

### Range and purpose of digital device use

The use of digital devices is now an essential part of adolescent life style. Adolescents regularly use computers to perform both scholastic as well as leisure activities [25]. In Korea, 60% of the population was reported as using smartphones in August 2012, only a few years after their introduction [26]. In the present study, while analyzing the different types of electronic devices used and the purpose for which they are used, almost 60% students used a smartphone and around two thirds used these devices for school projects. 43.6% of children use smartphones for gaming. Although excessive gaming has been shown to have detrimental health effects [13, 16], a recent review has concluded that the video games do not negatively impact adolescent academic performance in science, mathematics or reading [27].

### Digital device usage at night

The crispness of high-definition television screens, laptops and tablets can feel easier on the eyes as compared to older, less defined screens. Most digital screens are backlit and emit blue light or high-energy visible (HEV) light wavelengths. There is evidence that the eye is susceptible to blue light exposure, and that over a period of time the cumulative damage may increase the likelihood and severity of eye disorders (e.g. age-related macular degeneration and cataracts) [28]. Studies are also reporting the negative impact of smartphone usage on sleep. A decrease in melatonin secretion is attributed to the blue light exposure from smartphone displays. Yoshimura et al. have reported that the reduced viewing distance when lying down has a positive correlation to a poorer quality sleep (*R2 = 0.27 P < 0.05*), longer sleep latency (*R2 = 0.35, P < 0.05*) and lower sleep efficiency (*R2 = 0.38, P < 0.05*) [29]. In our study about one fifth of participants, 19.3% (111) used their smartphones at bedtime with lights switched off. We also observed that as age increased, the use of smartphones at bedtime with lights switched off also increased, with the ratio of those using smartphones to those not using them in the age group of 11–12 being approximately 1:10, compared with approximately 1:3 in the 16–17 age group. Studies have previously shown that this type of usage may lead to reduced sleep quality, potentially increasing the likelihood of experiencing other ocular pathologies later in life [10, 28, 29]. However, more studies are needed to establish a causal relationship between digital device usage and ocular diseases.

### Limitations of the study

Due to scarce literature on asthenopia and its correlation with digital device use in children, it is not possible to compare the results of this study directly with other reports. Our results need to be interpreted with caution. Firstly, data was acquired by the student’s self-answered questionnaire, and may therefore be subject to recall bias. Secondly, enrolment in this study was limited to students of urban private schools, and therefore results may not be representative of other populations. A broader study population (for instance including students of government schools or rural schools) may yield varied outcomes. Another limitation of the study was that the age groups were unequally distributed and were not age- or sex-matched. The reading distance in our study could have been measured more objectively using a measuring tape while asking the children to hold a digital device in their habitual position however manpower and time limitation need to be taken into account for taking such measurements in future studies. Despite our best efforts to exclude all causes of decreased vision and convergence insufficiency, existing visual conditions may still have acted as confounders on the prevalence of eyestrain in the population. Thus, recording both presenting and best-corrected visual acuity with the type of refractive error in future studies may help reduce the impact of this confounder. Despite the above limitations, our findings represent an important contribution to the literature, as they suggest that the current wave of digital development may have significant adverse ocular effects. Moreover, the results of this study add to the growing body of evidence investigating the adverse health effects of electronic media use among children.
